# Computer-assisted total knee arthroplasty using mini midvastus or medial parapatellar approach technique: A prospective, randomized, international multicentre trial

**DOI:** 10.1186/s12891-016-0872-7

**Published:** 2016-01-13

**Authors:** Peter Feczko, Lutz Engelmann, Jacobus J. Arts, David Campbell

**Affiliations:** Department Orthopaedic Surgery, Research School Capri, Maastricht University Medical Centre, P. Debyelaan 25, 6229 HX Maastricht, The Netherlands; Heinrich-Braun-Krankenhaus Zwickau, Städtisches Klinikum, Zwickau, Germany; Repatriation General Hospital, Adelaide, Australia

**Keywords:** Total knee arthroplasty, Navigation, Minimally invasive surgery, Blood loss, Accuracy

## Abstract

**Background:**

Despite the growing evidence in the literature there is still a lack of consensus regarding the use of minimally invasive surgical technique (MIS) in total knee arthroplasty (TKA).

**Methods:**

A prospective, randomized, international multicentre trial including 69 patients was performed to compare computer-assisted TKA (CAS-TKA) using either mini-midvastus (MIS group) or standard medial parapatellar approach (conventional group).

Patients from 3 centers (Maastricht, Zwickau, Adelaide) with end-stage osteoarthritis of the knee were randomized to either an MIS group with dedicated instrumentation or a conventional group to receive cruciate retaining CAS-TKA without patella resurfacing. The primary outcome was to compare post operative pain and range of motion (ROM). The secondary outcome was to measure the duration of surgery, blood loss, chair rise test, quadriceps strength, anterior knee pain, Knee Society Score (KSS),WOMAC scores, mechanical leg axis and component alignment.

**Results:**

Patients in the MIS group (3.97 ± 2.16) had significant more pain at 2 weeks than patients in the conventional group (2.77 ± 1.43) *p* = 0.003. There was no significant difference in any of the other primary outcome parameters. Surgery time was significantly longer (*p* < 0.001) and there were significantly higher blood loss (*p* = 0.002) in the MIS group as compared to the conventional group. The difference of the mean mechanical leg alignment between the groups was not statistically significant (–0.43° (95 % CI –1.50 – 0.64); *p* = 0.43).

There was no significant difference of component alignment between the two surgical groups with respect to flexion/extension (*p* = 0.269), varus/valgus (*p* = 0.653) or rotational alignment (*p* = 0.485) of the femur component and varus valgus alignment (*p* = 0.778) or posterior slope (*p* = 0.164) of the tibial component.

**Conclusion:**

There was no advantage of the MIS approach compared to a conventional approach CAS-TKA in any of the primary outcome measurements assessed, however the MIS approach was associated with longer surgical time and greater blood loss. MIS-TKA in combination with computer navigation is safe in terms of implant positioning.

**Trial registration number:**

ClinicalTrials.gov NCT02625311 8 December 2015

## Background

Total knee arthroplasty is a successful surgical treatment for debilitating osteoarthritis of the knee [1–3]. This intervention results excellent long-term survivorship [4–7] and marked improvement in functional capacity and quality of life of the patients [8] . However the conventional medial parapatellar approach is associated with local tissue disruption, interruption of neurovascular tissues, dislocation of the patella and the joint itself [9] resulting in a long hospital stay and long rehabilitation [10, 11]. To ameliorate these issues smaller incisions and muscle preserving approaches have been a prominent trend in total knee arthroplasty in more than 2 decades [12]. Five basic principles of the minimally invasive (MIS) techniques are described [13]: 1. Minimal interruption of nervous tissue and vascular supply. 2. Minimal dissection of muscles, tendons and ligaments. 3. Minimal resection of bone. 4. Minimal blood loss. 5. Minimal pain to the patient. The length of the incision is the least important aspect and should be long enough for the mobile-window technique. Based on the MIS principles, four techniques have become popular in clinical practice and research activities [14, 15]: the mini-subvastus approach [16], the mini-midvastus approach [17], the quadriceps-sparing approach [18, 19] and the mini medial parapatellar approach [20]. Comparative studies have not found a particular MIS approach to be superior or significantly better than anothers [21–23].

The short term results of MIS reported shorter length of hospital stay, better postoperative pain control, less blood loss, better quadriceps function and improved knee flexion compared to a conventional medial parapatellar approach [24–31]. However increased short term adverse event rates and less accuracy of implant position were also reported using MIS approaches [11, 32–36].

Other studies did not find statistical significant differences in pain, range of movement (ROM), Quadriceps strength, Knee Society Score (KSS) [37–40]. The results of meta-analyses are conflicting [28, 36, 41–45].

Computer navigation for total knee arthroplasty was first introduced in Europe in the 1990s, and there has been a widespread increase in its use throughout the world in the last decade. The proposed benefits of computer navigation for total knee arthroplasty include improved accuracy of both tibial and femoral component positioning and overall mechanical alignment [46]. Most studies comparing computer navigation with standard total knee arthroplasty have demonstrated a greater number of patients with coronal mechanical axis alignment within 3 of neutral in the navigation group. The outcome of a previous study showed that CAS-TKA reduced the overall rate of revision and the rate revision for loosening, but it has no effect on the short- and mid-term clinical outcomes [46].

We examined the synergy of combining computer-assisted surgery with a MIS approach with the hypothesis that computer assisted TKA improves component orientation and postoperative limb alignment that has been problematic in some non-computer assisted MIS-TKR studies [47–49]. We examined the hypothesis that minimally invasive, computer-assisted surgery would improve the short-term outcome without compromising the long term survivorship of TKA [50, 51].

The aim of our study was to perform a prospective, randomized multicentre trial to compare computer-assisted TKA using either a mini-midvastus (MIS group) or a medial parapatellar approach (conventional group).

The primary outcome was to compare postoperative pain and range of motion (ROM). The secondary outcome was to compare clinical data including duration of surgery, blood loss, chair rise, quadriceps strength, anterior knee pain, Knee Society Score (KSS) and WOMAC scores. Mechanical leg axis and component positioning was also evaluated on radiology and CT investigations respectively.

## Methods

### Trial design

A prospective, randomized, international multicentre trial including 69 patients was performed according to a standard protocol to compare computer-assisted TKA using either a mini-midvastus (MIS group) or a medial parapatellar approach (conventional group). Patients in 3 centers (Maastricht, Zwickau, Adelaide) with end-stage osteoarthritis of knee were randomized to either an MIS group with dedicated instrumentation or a conventional group.

### Ethics

Ethical approval of all centres had been obtained from the local ethical committee of Maastricht (MEC 04-105), Adelaide (RGH 10/04) and Zwickau (EK-MPG-0603) as part of the research program “A prospective comparative, randomized study comparing the MIS computer navigated total knee arthroplasty vs. conventional computer navigated total knee using the Scorpio CR fixed bearing knee and the Stryker navigation system”.

Trial Registration Number: ClinicalTrials.gov NCT02625311 8 December 2015.

### Participant selection and consent

Patients were randomized (random permuted blocks of 4) in either the MIS group or the conventional group. A written informed consent was obtained from all participants.

Seventy six participants were included for the study. There were 4 intra-operative exclusions: 3 patients due to problems with the navigation trackers and one patient due to fracture of the tibial plateau.

Three patients were lost to follow up due to unwillingness of the participants. Sixty nine patients completed the 6 months study period.

All cases were performed by a single surgeon in each of the three centres. Before the study, the surgeons participated in training involving multiple cases of cadaver prosthetic implants using the navigation system with the MIS approach and a minimum of ten clinical cases.

### Inclusion and exclusion criteria

Patients between 45 and 75 years of age who had an established diagnosis of knee osteoarthritis requiring primary total knee replacement. Exclusion criteria included previous cruciate ligament reconstruction, correction osteotomy of the tibia, patellectomy, BMI greater than 30, flexion contracture greater than 15°, varus or valgus deformity greater than 15°, medio-lateral instability greater than 10° and active inflammation or infection of the knee. In addition, patients were excluded if they were pre-operatively considered to require patellar surface implantation.

### Interventions (operative procedure)

The Stryker (Stryker Howmedica Osteonics, Allendale, NJ USA) Navigation System II, version 3.1 was used in all cases. This is an image free, active, cordless system. All knee surgeries were performed using a tourniquet. In the MIS group a 10 cm incision with a flexed knee, mini-midvastus approach was applied per standard protocol and with dedicated instrumentation. In cases where conventional surgical technique was used, a medial parapatellar approach was applied. The navigation system trackers were then attached to the surface of the femur and the tibia. The hip joint rotation center and the center of the ankle joint were established as reference points for leg axis. The rotational position of the femoral component was determined by using the *Whiteside’s line* and the transepicondylar line (*TEA*). Tibial rotation was assessed based on the relative positions of the centre of the ankle joint and the medial one third of tibial tubercle. The navigation was accorded with the neutral mechanical axis of the extremity with tibial slope fixed at four degrees. Each resurfacing plane angle was instrumented with dedicated navigation cutting guides and checked with the navigation system following the osteotomy. With both techniques, after determining proper prosthetic size, the collateral ligaments were balanced as required based on ligament tension assessed during functional testing of the prosthetic implant. Patellar surface implantation was not performed. The femoral component was implanted without cementing, whereas the tibial component was cemented with Simplex P (Stryker Howmedica Osteonics, Allendale, NJ USA) containing antibiotics. In each case, a Scorpio (Stryker Howmedica Osteonics, Allendale, NJ USA) CR fixed bearing implant was used without patellar surface implantation.

### Outcome measurements

Clinical outcomes were assessed by a blinded independent examiner. All outcome parameters were assessed preoperative and postoperative at 7 weeks, 3 and 6 months. Pain and ROM were also measured weekly between postoperative weeks two to seven.

The primary outcome was to compare postoperative pain and range-of-motion (ROM) of both groups. Pain was assessed using a Likert score [52]. Range of motion (ROM) was measured using a goniometer according to the technique described Norkin [53]. Intra-tester and inter-tester reliability was described by Brosseau [54], the reproducibility by Lenssen [55].

The secondary outcome was to compare duration of surgery, blood loss, chair rise test, quadriceps strength test, anterior knee pain, KSS and WOMAC score. Duration of surgery was measured in minutes from skin incision to closure of the wound. Blood loss was measured intra-operative and during the first 24 h postoperative. All data was recorded in ml. Chair rise test was assessed according to the description of Jones [56]. The patients were sitting on a stool with the hip and knee in 90° of flexion. The patients had to stand up from the stool without using their arms. The test was repeated five times. Quadriceps strength test (fair/good – can break/good – can’t break) [57, 58] and anterior knee pain arising from a chair (yes or no) was assessed by the method described by Insall et al. [58] Knee Society Scores [58] and WOMAC scores [59] were also measured.

The primary hypothesis was that those patients who underwent MIS would benefit from less postoperative pain and higher ROM. The secondary hypothesis was that the use of computer navigation allows MIS-TKA to be performed without increased risk of limb mal-alignment more than 2° and outliners in component positioning.

### Radiological evaluations

The lower limb mechanical axis was measured on long standing radiographs preoperatively and at 3 months postoperatively [60]. Outliers were defined as a coronal mechanical leg alignment of more than 2° from neutral. CT scan was performed three months postoperatively with analysis of component alignment determined by the Perth protocol [61]. The position of femoral component was determined in sagittal, coronal and transverse planes, the tibial component was determined in sagittal and coronal planes. Outliners were defined as the component were positioned more than 2° different than the planned position. Mean values were used for further analyses.

### Statistics

Descriptive statistics were used to summarize the data. Categorical data were analyzed using Pearson Chi square test, likelihood Chi square tests or Fisher’s Exact tests. For continuous data Student’s *t*-test, or two-way ANOVA was used. Analyses were performed using SPSS v19.0. *P*-values < 0.05 were considered statistically significant.

Boxplots represent 10, 25, 50, 75, and 90 % of data. Outliers are shown as dots. Means are not presented in the boxplots.

## Results

### Demographics

There was no significant difference between the two surgical groups with respect to sex, age, BMI, side of operation or primary diagnosis at *p* > 0.05 (Table 1).

**Table 1:** Demographics & baseline characteristics

	MIS (*n* = 36)	Conventional (*n* = 33)	*p*-value
Sex (F/M)^e^	23/13	22/11	0.81^b^
Age (years)^f^	65.14 ± 8.35	64.88 ± 6.78	0.89^a^
BMI (kg/m^2^)^f^	28.26 ± 2.81	28.56 ± 2.93	0.67^a^
Side of operation (R/L)^e^	22/14	18/15	0.95^b^
Diagnosis (Primary/Posttraumatic OA)^e^	35/1	33/0	0.52^d^
Chair rise (yes/no)^e^	26/10	25/8	0.74^b^
Anterior knee pain (yes/no)^e^	27/9	26/7	0.71^b^
Quadriceps strength (fair/good – can break/good – can’t break)^e^	1/16/19	0/21/12	0.21^c^
KSS^f^	108.91 ± 26.42	99.36 ± 25.02	0.13^a^
WOMAC^f^	78.08 ± 12.92	76.39 ± 10.56	0.56^a^

^a^Student’s *t*-test, ^b^Pearson *χ* ^2^-test, ^c^likelihood ratio *χ* ^2^-test and ^d^Fisher’s Exact test

^e^Value are numbers. ^f^Values are mean ± sd

### Primary outcomes

#### Pain

There was a statistically significant difference in pain scores between groups (two-way ANOVA, F(1) = 13.32; *p* = 0.003). Post-hoc comparison of between group differences showed a 1.20 (95 % CI 0.27 – 2.12; *p* = 0.01) points difference in favor of the conventional group at week 2 (MIS 3.97 points ± 2.16 vs. conventional 2.77 points ± 1.43). At the other time points, no differences in pain scores between both groups were found (Table 2).

**Table 2:** Pain. (Likert scale)

	MIS (*n* = 36)	Conventional (*n* = 32)	Difference (95 % CI) & *p*-value^a^
Surgery time (min)	134.53 ± 21.85	103.56 ± 14.93	30.97 (21.79 – 40.14); *p* < 0.001
Intraoperative blood loss (ml)	73.06 ± 99.82	58.06 ± 79.22	14.99 (-29.49 – 59.47); *p* = 0.50
Postoperative blood loss first 24 h (ml)	726.11 ± 471.63	411.09 ± 324.76	315.02 (116.50 – 513.54); *p* = 0.002

^a^Student’s *t*-test

### Range of motion

No differences in range of motion between groups were found at the different time points (two-way ANOVA, F(1) = 0.73; *p* = 0.12) (Table 3).

**Table 3:** Range of motion

	Time					
	Week 2	Week 3	Week 4	Week 5	Week 6	Week 7
MIS	3.97 ± 2.16	2.97 ± 1.68	3.19 ± 1.43	2.72 ± 1.47	2.20 ± 1.23	1.97 ± 1.10
Conventional	2.77 ± 1.43	2.55 ± 1.71	2.62 ± 1.66	2.10 ± 1.11	1.86 ± 1.09	1.81 ± 1.13
*p*-value						0.003^a^

^a^Two-way ANOVA. Represents between group *p*-value for factor ‘treatment’ (F(1) = 13.32). Post-hoc comparison of between group differences showed a 1.20 (95 % CI 0.27 – 2.12; *p* = 0.01) points difference in favor of the conventional group at week 2

### Secondary outcomes

#### Duration of surgery and blood loss

Surgery time was significantly longer (30.97 min (95 % CI 21.79 – 40.14); *p* < 0.001) in the MIS group (134.53 ± 21.85) as compared to the conventional group (103.56 ± 14.93) (Table 4). There was no significant difference 14.99 ml (95 % CI 29.49 – 59.47); *p* = 0.50) between MIS group (73.06 ± 99.82) and conventional group (58.06 ± 79.22) in intra-operative blood loss. However, the first 24 h blood loss was significantly higher 315.02 ml (95 % CI 116.50 – 513.54); *p* = 0.002) in the MIS group (726.11 ml ± 471.63) as compared to the conventional group (411.09 ml ± 324.76) (Table 4).

**Table 4:** Surgery time, intraoperative blood loss & postoperative blood loss (first 24 h)

	Time							
	Week 2	Week 3	Week 4	Week 5	Week 6	Week 7	3 months	6 months
MIS	81.97 ± 16.20	90.97 ± 13.57	92.50 ± 15.49	96.04 ± 14.47	97.69 ± 13.51	97.00 ± 12.40	103.57 ± 13.15	106.67 ± 12.91
Conventional	79.35 ± 14.19	90.65 ± 10.78	93.85 ± 10.49	96.75 ± 8.60	98.40 ± 8.86	101.12 ± 9.16	103.77 ± 10.74	105.97 ± 11.58
*p*-value								0.12^a^

^a^Two-way ANOVA. Represents between group *p*-value for factor ‘treatment’ (F(1) = 0.73). No difference between groups

### Chair rise, quadriceps strength and anterior knee pain

At 7 weeks, 3 months and 6 months follow-up, no differences between types of surgery in the ability to rise from a chair (yes/no) (*p* = 0.56, *p* = 0.38, and *p* = 0.10 for 7 weeks, 3 months, and 6 months, respectively; Table 5), between types of surgery in quadriceps strength (fair/good – can break/good – can’t break) (*p* = 0.60, *p* = 0.30, and *p* = 0.69 for 7 weeks, 3 months, and 6 months, respectively; Table 6), and between types of surgery in the presence of anterior knee pain (yes/no) were found (*p* = 0.59, *p* = 0.66, and *p* = 0.11 for 7 weeks, 3 months, and 6 months, respectively; Table 7).

**Table 5:** Chair rise test (yes/no)

	Time		
	7 weeks	3 months	6 months
MIS	24/12	26/9	26/7
Conventional	22/8	25/5	28/2
*p*-value	0.56^a^	0.38^a^	0.10^b^

^a^Pearson *χ* ^2^-test. ^b^Fisher’s exact test

**Table 6:** Anterior knee pain (yes/no)

	Time		
	7 weeks	3 months	6 months
MIS	9/26	2/32	6/27
Conventional	6/24	3/27	1/29
*p*-value	0.59^a^	0.66^b^	0.11^b^

^a^Pearson *χ* ^2^-test. ^b^Fisher’s Exact test

**Table 7:** Quadriceps strength (fair/good – can break/good – can’t break)

	Time		
	7 weeks	3 months	6 months
MIS	1/17/18	1/14/20	0/8/25
Conventional	2/16/12	0/17/13	0/6/24
*p*-value	0.60^a^	0.30^a^	0.69^a^

^a^Likelihood ratio *χ* ^2^-test

### Functional scores: KSS and WOMAC

At 7 weeks, 3 months and 6 months follow-up, there were no differences between groups in functional scores (KSS score (two-way ANOVA, F(1) = 0.43; *p* = 0.51, Table 8) and WOMAC (Two-way ANOVA; F(1) = 0.005; *p* = 0.94, Table 9).

**Table 8:** KSS score

	Time		
	7 weeks	3 months	6 months
MIS	141.68 ± 29.61	156.11 ± 31.12	168.15 ± 29.61
Conventional	144.03 ± 22.89	157.75 ± 27.98	171.87 ± 19.05
*p*-value			0.51^a^

^a^Two-way ANOVA. Represents between group *p*-value for factor ‘treatment’ (F(1) = 0.43). No difference between groups

**Table 9:** WOMAC score

	Time		
	7 weeks	3 months	6 months
MIS	47.39 ± 15.41	15.67 ± 2.52	18.15 ± 16.51
Conventional	48.48 ± 13.63	18.00 ± 2.83	15.57 ± 12.58
*p*-value			0.94^a^

^a^Two-way ANOVA. Represents between group *p*-value for factor ‘treatment’ (F(1) = 0.005). No difference between groups

### Mechanical leg axis

**Fig. 1:**
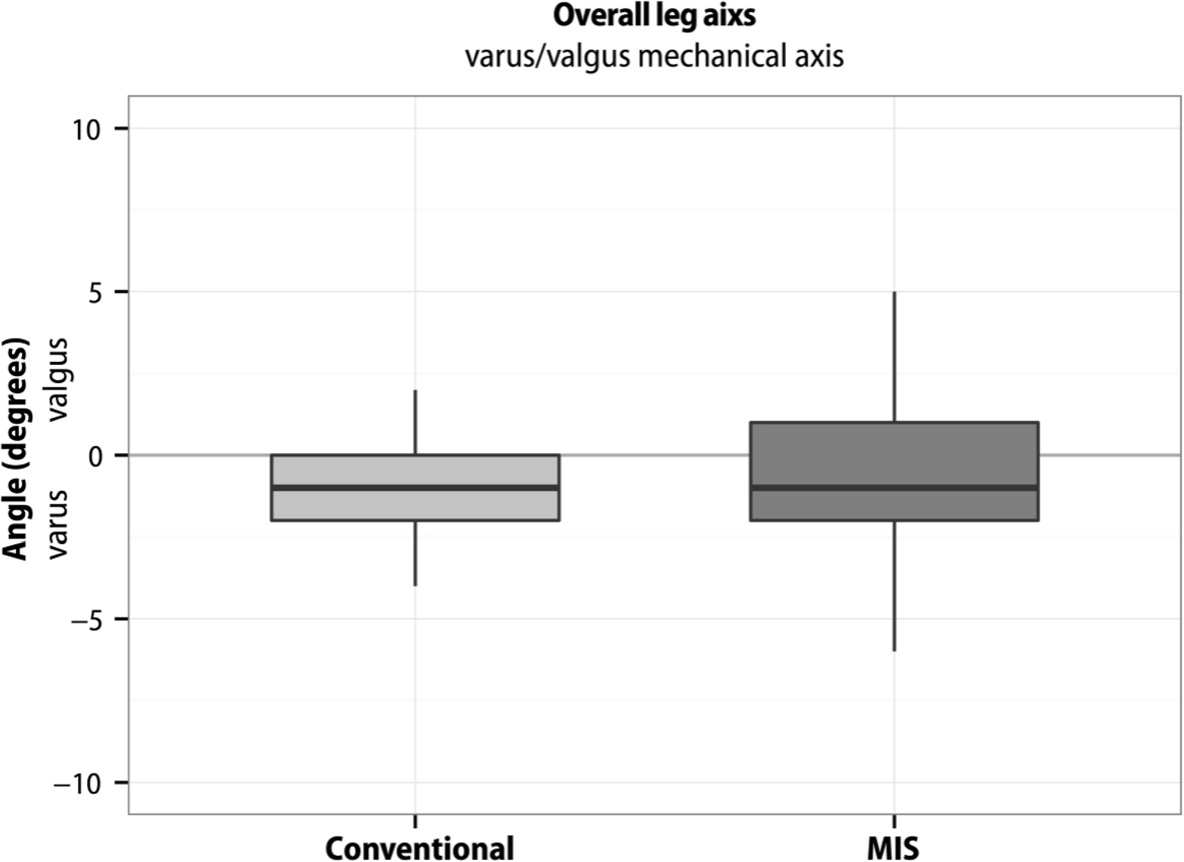
Boxplot of the mechanical leg axis (degrees) for MIS and conventional TKA groups. The mean mechanical leg axis in the conventional group was 0.97° ± SD 1.87°, in the MIS group 0.54° ± SD 2.53°. The difference of the mean mechanical leg alignment between the groups was not statistically significant (‐0.43° (95 % CI ‐1.50 - 0.64); *p* = 0.43)

Limb alignment coronal axis was achieved within a target of 2° equally in both groups (75 % of patients in the MIS group and 78.7 % of patients in the conventional approach group). The mean mechanical leg axis in the conventional group was 0.97° ± SD 1.87°, in the MIS group 0.54° ± SD 2.53° (Fig. 1). The difference of the mean mechanical leg alignment between the groups was not statistically significant (‐0.43° (95 % CI ‐1.50 - 0.64); *p* = 0.43).

### Component positioning

**Fig. 2:**
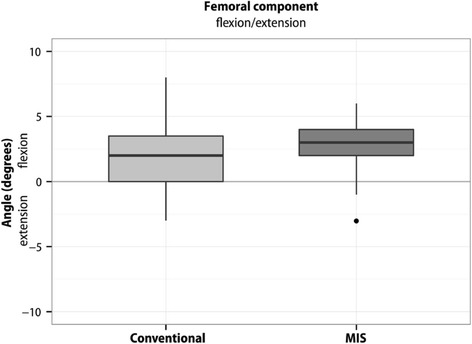
Flexion/extension position of the femoral component. The flexion position in the conventional group was 1.94° ± SD 2.54°, in the MIS group 2.77° ± SD 2.09°. The difference between the two surgical groups was (‐0.83° (95 % CI ‐2.32 - 0.67); *p* = 0.27). Outliers are shown as dots

**Fig. 3:**
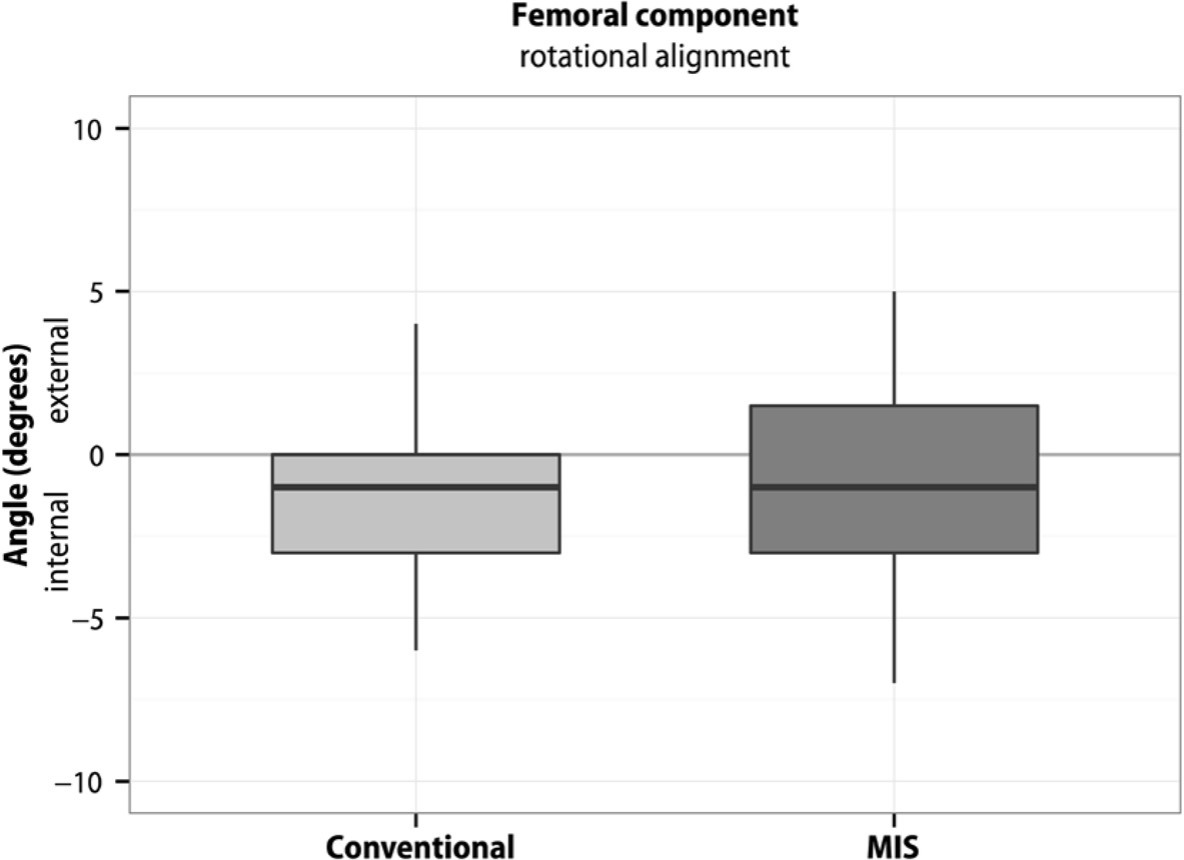
Rotational position of the femoral component. The rotational position in the conventional group was -1.00° ± SD 2.22°, in the MIS group -0.55° ± SD 2.74°. The difference between the two surgical groups was (‐0.45° (95 % CI ‐1.74 - 0.83); *p* = 0.49). Outliers are shown as dots

**Fig. 4:**
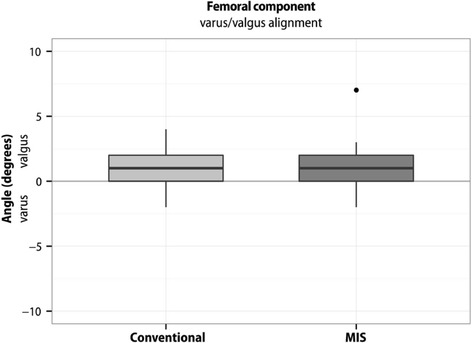
Varus/valgus position of the femoral component. The varus position in the conventional group was 0.71° ± SD 1.64°, in the MIS group 0.89° ± SD 1.68°. The difference between the two surgical groups was (‐0.18° (95 % CI ‐0.99 - 0.62); *p* = 0.65). Outliers are shown as dots

**Fig. 5:**
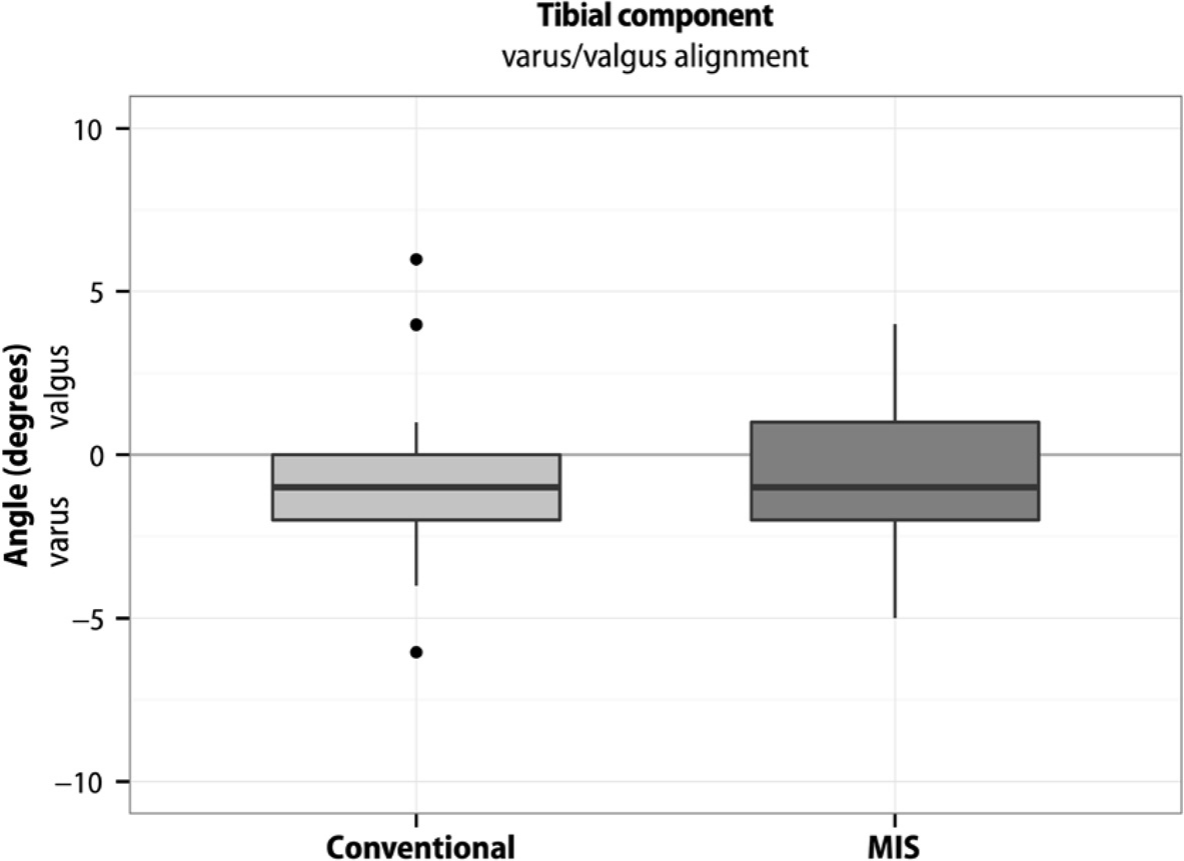
Varus/valgus position of the tibial component. The varus position in the conventional group was -0.97° ± SD 2.30°, in the MIS group -0.81° ± SD 2.25°. The difference between the two surgical groups was (‐0.16° (95 % CI ‐1.26 - 0.95); *p* = 0.78). Outliers are shown as dots

**Fig. 6:**
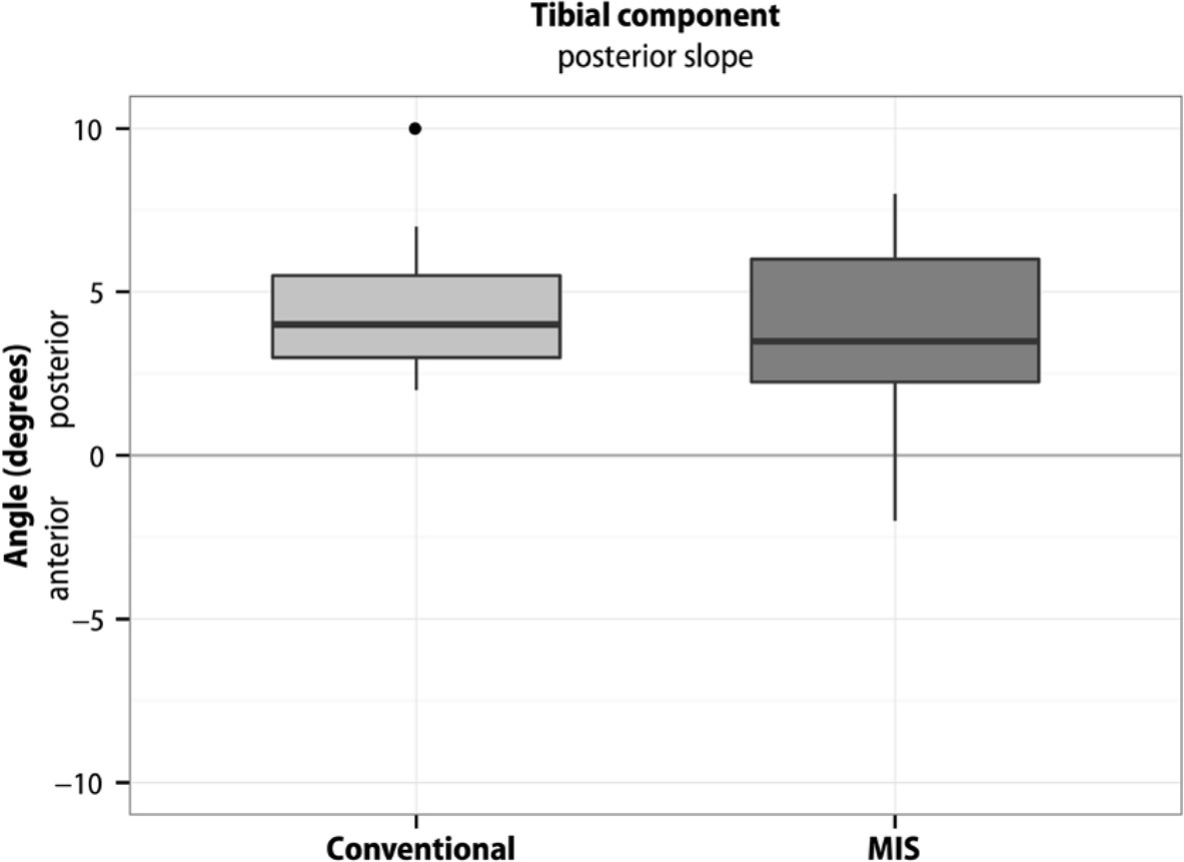
Posterior slope of the tibial component. The posterior slope in the conventional group was 4.63° ± SD 1.86°, in the MIS group 3.63° ± SD 2.61°. The difference between the two surgical groups was (1.00° (95 % CI ‐0.43 - 2.42); *p* = 0.16). Outliers are shown as dots

There was no significant difference between the two surgical groups with respect to flexion/extension (‐0.83° (95 % CI ‐2.32 - 0.67); *p* = 0.27), rotational alignment of the femur component (‐0.45° (95 % CI ‐1.74 - 0.83); *p* = 0.49) or varus/valgus (‐0.18° (95 % CI ‐0.99 - 0.62); *p* = 0.65) and varus/valgus alignment (‐0.16° (95 % CI ‐1.26 - 0.95); *p* = 0.78) or posterior slope (1.00° (95 % CI ‐0.43 - 2.42); *p* = 0.16) of the tibial component (Figs. 2, 3, 4, 5 and 6).

## Discussion

The primary hypothesis that patients of the MIS group would have less pain and better early ROM was not confirmed by the results of this study.

Most studies comparing MIS-TKA with conventional TKA report lower visual analog pain score [24, 25, 36, 39, 43, 44] and better range of motion [24, 36, 39, 43, 44] in the MIS group in the early postoperative period (2-6 weeks). However, the same studies following this variable closely over time report little or no difference between the two groups in subsequent follow-ups (3-6 months). Heekin [40] reported inconsistent pattern in pain and ROM, while other study [41] and meta-analysis [44] found no difference in any of the time points.

The mini-midvastus CAS-TKA resulted in significantly more blood loss as well as an elongated surgery time. Nearly all studies and meta analyses report significant longer duration of surgery in the MIS approach in comparison with medial parapatellar approach. There is no agreement in previous studies in term of blood loss. Some authors reported significant less blood loss [10, 11, 32, 41, 43, 44] in favor of the minimally invasive approach, while others did not find any differences between techniques [28, 34, 37–39, 42].

One of the most important potential benefits of applying the MIS technique is the ability to avoid manipulation of the extensor apparatus and theoretically a shorter recovery time for quadriceps muscle strength was expected [4, 5, 11]. However, we found no significant difference between the groups quadriceps muscle strength assessments, chair rise tests or anterior knee pain. The results measuring quadriceps strength in other studies are also contradictive [25, 31, 34, 44]. There is very few data available in anterior knee pain comparing minimally invasive approach with medial parapatellar approach [23].

The MIS surgery also failed in our study to generate clear advantages in KSS and WOMAC scores. In both groups, there was a marked postoperative improvement of both KSS and WOMAC scores compared to preoperative values. However, no difference was found between the two groups postoperative values. Similar results are shown in the most publications included meta-analyses [24, 28, 32, 34, 37–40, 42].

The secondary hypothesis was that the use of computer navigation allows MIS-TKA to be performed without increased risk of limb mal-alignment more than 2° and outliners in component positioning [20] was confirmed. The MIS technique did not result in implant mal-positioning. Component and limb alignment was also comparable between the two surgical approaches and we did not observe an increased incidence of mal-alignment that has been associated with the restricted access and visualization of the MIS approach [19]. The use of computer assisted navigation can be a useful adjunct to the MIS technique and our results can be confirmed by other authors [46–51].

## Conclusion

When comparing the relative merits of the minimally invasive and the conventional approaches we could not confirm any of the short term benefits expected from the minimally invasive technique. There was no advantage of the MIS approach compared to a conventional approach CAS-TKA in any of the primary outcome measurements assessed, however the MIS approach was associated with longer surgical time and greater blood loss. MIS-TKA in combination with computer navigation is safe in terms of implant positioning.

### Limitation of the study

The number of patients in our study is rather low and the sample size calculation is missing, however the findings of this study is in line with the findings of previous researches.

Although all of the three surgeons were experienced, high-volume knee surgeons, they had much more experience in medial parapatellar approach than mini-midvastus approach, since the conventional approach was used routinely in all clinics.

The authors did not determine the rotational position of the tibial component since it was not a primary objective of the study and there was no statistical significant difference between groups in terms of component positions in other planes. The rotational alignment of the tibial tray might be theoretically different between groups, however is not in line with the findings of previous studies.
